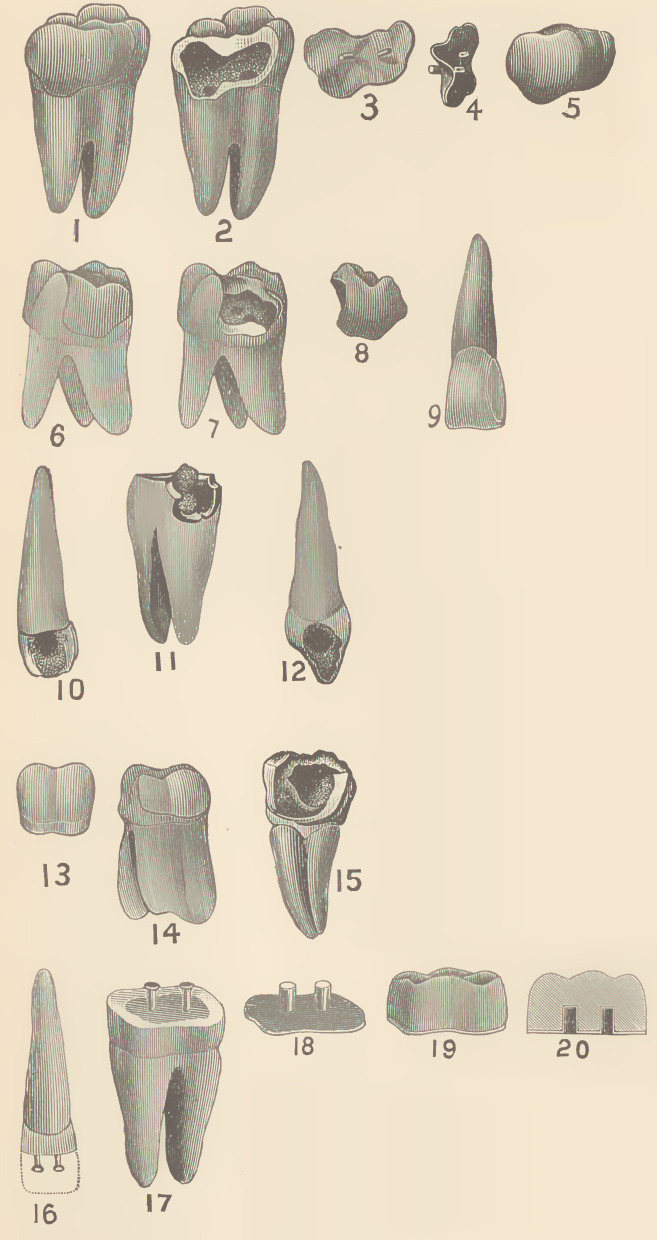# Metallic Enamel Sections

**Published:** 1887-02

**Authors:** C. H. Land

**Affiliations:** Detroit, Mich.


### METALLIC ENAMEL SECTIONS. A NEW SYSTEM FOR FILLING TEETH.

BY DR. C. H. LAND, DETROIT, MICH.

In the July number of the INDEPENDENT PRACTITIONER, a description is given of my new process of coating badly decayed teeth. In addition to this, I have devised a means of filling teeth with prepared sections of porcelain, or it may be designated as a system of partial crown work. By reference to the engraving, Figs. 2, 7, 10, 12 and 15, there will be seen characteristic conditions of decay suitable for this class of work. Figs. 2 and 7 are the prepared cavities on anterior sides of molars. The manner of procedure is to burnish a thin piece of annealed platinum plate into the cavity. This takes a perfect impression of its outlines. The surplus edges are trimmed off and platinum pins attached, using pure gold leaf for solder. See Figs. 3 and 4. The pins serve as a fastening, both to secure the completed section in place and as retainers for the porcelain body. Figs. 5 and 8 illustrate the completed sections, showing contour of the original shape of the lost portion of the natural tooth. Figs. 1 and 6 are prepared sections cemented in place.

Having secured the prepared sections as shown in Figs. 3 and 4, porcelain paste or body is built upon them and carved so as to imitate the original contour of the lost portion of the tooth, as shown in Figs. 5 and 8. They are then placed on a bed of silex and fused in a gas furnace. This requires twenty minutes for the first biscuit, and fifteen for the second. When completed, they will be a reproduction in porcelain of the lost parts of the natural organs, resembling nature perfectly, both in color and shape. They are then cemented in the cavity, either with gutta-percha filling or oxy-phosphate cement. When the anterior side of a molar or bicuspid is decayed, as shown in Figs. 10 and 15, the enamel front or veneer, 13, is added to the porcelain body, and when completed it will appear as shown in Fig.

14. This veneer serves as a ready and efficient means of securing the proper shade and contour of each class of teeth. To those who are not familiar with the use of a gas furnace this class of work may seem difficult, but a little experience with the modern appliances now within the reach of every dentist, makes the operation a comparatively simple and easy one. Figs. 17, 18, 19 and 20 are a modification. Fig. 17 represents a tooth filled with gold, having two pins attached. Fig. 18 is a platinum disk, with tubes adjusted to correspond to the position of the pins in Fig. 17. Porcelain body is built about the tubes, and when fused in the furnace the whole will form a porcelain crown as shown in Fig. 19. Fig. 20 illustrates the relative position of the tubes, which are designed to form countersinks for the pins in Fig. 17. When cemented in place, it makes a very durable and beautiful piece of work. Fig. 16 is an incisor constructed in a similar manner. From this will be seen the great advantage of being able to have the porcelain in a plastic state, as it enables the dentist to perfectly adapt the form of each peculiar case with the utmost precision, and this could not be so admirably done with manufactured crowns.

In bringing this new mode of practice to the notice of the dental profession, I wish to call especial attention to the large amount of tooth substance preserved. In nearly all the modern systems of crown-work there seems to be too much good tooth material cut away, and I think a careful investigation will demonstrate this new process to be far superior, making it possible to save the greater portion of the crown, it not being necessary to cut beneath the gum. In nearly every case, sufficient tooth substance can be retained to preserve the pulp alive, and when the teeth are devitalized the major portions of the crown can be left intact, serving for retaining purposes and making it unnecessary, in the majority of cases, to resort to screws or posts. Fig. 16 illustrates a section of porcelain adjusted to a central incisor which, when carefully done, makes a very acceptable piece of work. Although the joint may sometimes be conspicuous, it is not nearly as much so as a glaring piece of gold.

The numerous opportunities presented in which this porcelain process will prove to be of great value, is almost without limit, and has enabled me to practice dentistry on an entirely new basis, so that to-day I can say to my patients that their teeth can be perfectly restored, both in appearance and usefulness, no matter how badly they are decayed. No pulps will be destroyed, and very little tooth substance need be cut away. The use of the rubber dam is largely dispensed with; there are no long and tedious malleting operations in large gold fillings, and no use for amalgam, yet the teeth can be perfectly restored in shape, color and size, with very little pain or fatigue either to the operator or patient.

As my labors for a higher art belong in equal proportion to my fellow co-laborers, and any improvements which I may devise will be presented to them free from the annoyance of patent rights, I take much pleasure in publishing these facts, hoping that they will be appreciated by my brethren as at least one step in the advancement of dental art.

## Figures and Tables

**Figure f1:**